# Correction to: Impact of the Covid-19 pandemic on primary care utilization: evidence from Sweden using national register data

**DOI:** 10.1186/s13104-022-05972-x

**Published:** 2022-02-22

**Authors:** Björn Ekman, Eva Arvidsson, Hans Thulesius, Jens Wilkens, Olof Cronberg

**Affiliations:** 1 Department of Clinical Sciences, Malmö, Lund University, Jan Waldenströms gata 35, 202 05 Malmö, Sweden; 2 Futurum, Region Jönköping’s County, Jönköping, Sweden; 3 School of Health and Welfare, Jönköping University, Jönköping, Sweden

## Correction to: BMC Research Notes (2021) 14:424 https://doi.org/10.1186/s13104-021-05839-7

Following the publication of the original article [1], the authors informed us that a typographical error had occurred during the final stages of processing: the thousand comma-separators in Table 1 had inadvertently been misplaced.

**Table 1:** Primary care consultations by type and year, selected regions

Consultation type	Year	
	2019 N %	2020 N %
Clinic visits	10,499,364	8,819,487
	86.9	81.3
Telemedicine contacts	320,873	269,609
	2.7	2.5
Home visits	159,844	171,380
	1.3	1.6
Telephone/letter contacts	1,099,187	1,587,442
	9.1	14.6
Total	12,079,268	10,847,918

Source: VTDB 2019 and 2020

The correct table is included in this Correction as well as the original article, which has already been updated.
